# CEB Improves Model Robustness

**DOI:** 10.3390/e22101081

**Published:** 2020-09-25

**Authors:** Ian Fischer, Alexander A. Alemi

**Affiliations:** Google Research, Mountain View, CA 94043, USA; alemi@google.com

**Keywords:** information theory, information bottleneck, machine learning

## Abstract

Intuitively, one way to make classifiers more robust to their input is to have them depend less sensitively on their input. The Information Bottleneck (IB) tries to learn compressed representations of input that are still predictive. Scaling up IB approaches to large scale image classification tasks has proved difficult. We demonstrate that the Conditional Entropy Bottleneck (CEB) can not only scale up to large scale image classification tasks, but can additionally improve model robustness. CEB is an easy strategy to implement and works in tandem with data augmentation procedures. We report results of a large scale adversarial robustness study on CIFAR-10, as well as the ImageNet-C Common Corruptions Benchmark, ImageNet-A, and PGD attacks.

## 1. Introduction

We aim to learn models that make meaningful predictions beyond the data they were trained on. Generally we want our models to be robust. Broadly, robustness is the ability of a model to continue making valid predictions as the distribution the model is tested on moves away from the empirical training set distribution. The most commonly reported robustness metric is simply test set performance, where we verify that our model continues to make valid predictions on what we hope represents valid draws from the same data generating procedure as the training set.

Adversarial attacks test robustness in a worst case setting, where an attacker [1] makes limited targeted modifications to the input that are as fooling as possible. Many adversarial attacks have been proposed and studied (e.g., Szegedy et al. [1], Carlini and Wagner [2,3], Kurakin et al. [4], Madry et al. [5]). Most machine-learned systems appear to be vulnerable to adversarialexamples. Many defenses have been proposed, but few have demonstrated robustness against a powerful, general-purpose adversary [3,6]. Recent discussions have emphasized the need to consider forms of robustness besides adversarial [7]. The Common Corruptions Benchmark [8] measures image models’ robustness to more mild real-world perturbations. Even these modest perturbations can fool traditional architectures.

One general-purpose strategy that has been shown to improve model robustness is data augmentation [9,10,11]. Intuitively, by performing modifications of the inputs at training time, the model is prevented from being too sensitive to particular features of the inputs that do not survive the augmentation procedure. We would like to identify complementary techniques for further improving robustness.

One approach is to try to make our models more robust by making them less sensitive to the inputs in the first place. The goal of this work is to experimentally investigate whether, by systematically limiting the complexity of the extracted representation using the Conditional Entropy Bottleneck (CEB), we can make our models more robust in all three of these senses: test set generalization (e.g., classification accuracy on “clean” test inputs), worst-case robustness, and typical-case robustness.

This paper is primarily empirical. We demonstrate:

- CEB models are easy to implement and train.
- CEB models show improved generalization performance over deterministic baselines on CIFAR10 and ImageNet.
- CEB models show improved robustness to untargeted Projected Gradient Descent (PGD) attacks on CIFAR10.
- CEB models trained on ImageNet show improved robustness on the ImageNet-C Common Corruptions Benchmark, the ImageNet-A Benchmark, and targeted PGD attacks.
- CEB models trained on ImageNet show improved calibration on the ImageNet validation set and on ImageNet-C.

We also show that adversarially-trained models *fail* to generalize to attacks they were not trained on, by comparing the results on L${}_{2}$ PGD attacks from Madry et al. [5] to our results on the same baseline architecture. This result underscores the importance of finding ways to make models robust that do not rely on knowing the form of the attack ahead of time. Finally, for readers who are curious about theoretical and philosophical perspectives that may give insights into why CEB improves robustness, we recommend Fischer [12], which introduced CEB, as well as Achille and Soatto [13], Achille and Soatto [14], and Pensia et al. [15].

## 2. Materials and Methods

### 2.1. Information Bottlenecks

The Information Bottleneck (IB) objective [16] aims to learn a stochastic representation Z∼p(z|x) of some input *X* that retains as much information about a target variable *Y* while being as compressed as possible. The objective:

(1) $$IB\equiv \max_{p(z|x)}I\left(Z;Y\right)-\sigma \left(-\rho \right)I\left(Z;X\right),$$

uses a Lagrange multiplier σ(−ρ) to trade off between the relevant information (I(Z;Y)) and complexity of the representation (I(Z;X)). The IB objective is ordinarily written with a Lagrange multiplier β≡σ(−ρ) with a natural range from 0 to 1. Here we use the sigmoid function: $\sigma \left(-\rho \right)\equiv \frac{1}{1+e^{\rho }}$ to reparameterize in terms of a control parameter ρ on the whole real line. As ρ→∞ the bottleneck turns off.

Because *Z* depends only on *X* (Z←X↔Y), *Z* and *Y* are independent given *X*:

(2) I(Z;X,Y)=I(Z;X)+I(Z;Y|X)=I(Z;Y)+I(Z;X|Y).

This allows us to write Equation (1) in an equivalent form:

(3) $$\max_{Z}I\left(Z;Y\right)-e^{-\rho }I\left(Z;X|Y\right).$$

Just as the original IB objective (Equation (1)) admits a natural variational lower bound [17], so does this form. We can variationally lower bound the mutual information between our representation and the targets with a variational decoder q(y|z):

(4) $$\begin{array}{cc} I(Z;Y)= & E_{p(x,y)p(z|x)}\left[\log\frac{p(y|z)}{p(y)}\right]\\ \geq & H\left(Y\right)+E_{p(x,y)p(z|x)}\left[\log q(y|z)\right]. \end{array}$$

While we may not know H(Y) exactly for real world datasets, in the IB formulation it is a constant outside of our control and so can be dropped in our objective. We can variationally upper bound our residual information:    

(5) $$\begin{array}{cc} I(Z;X|Y)= & E_{p(x,y)p(z|x)}\left[\log\frac{p(z|x,y)}{p(z|y)}\right]\\ \leq & E_{p(x,y)p(z|x)}\left[\log\frac{p(z|x)}{q(z|y)}\right], \end{array}$$

with a variational class conditional marginal q(z|y) that approximates ∫dxp(z|x)p(x|y). Putting both bounds together gives us the Conditional Entropy Bottleneck objective [12]:

(6) $$\min_{p(z|x)}E_{p(x,y)p(z|x)}\left[-\log q\left(y|z\right)+e^{-\rho }\log\frac{p(z|x)}{q(z|y)}\right].$$

Compare this with the Variational Information Bottleneck (VIB) objective [17]:

(7) $$\min_{p(z|x)}E_{p(x,y)p(z|x)}\left[-\log q\left(y|z\right)-\sigma \left(-\rho \right)\log\frac{p(z|x)}{q(z)}\right].$$

The difference between CEB and VIB is the presence of a class conditional versus unconditional variational marginal. As can be seen in Equation (5), using an unconditional marginal provides a looser variational upper bound on I(Z;X|Y). CEB (Equation (6)) can be thought of as a tighter variational approximation than VIB (Equation (7)) to Equation (3). Since Equation (3) is equivalent to the IB objective (Equation (1)), CEB can be thought of as a tighter variational approximation to the IB objective than VIB.

### 2.2. Implementing a CEB Model

In practice, turning an existing classifier architecture into a CEB model is very simple. For the stochastic representation p(z|x) we simply use the original architecture, replacing the final softmax layer with a dense layer with *d* outputs. These outputs are then used to specify the means of a *d*-dimensional Gaussian distribution with unit diagonal covariance. That is, to form the stochastic representation, independent standard normal noise is simply added to the output of the network (z=x+ϵ). For every input, this stochastic encoder will generate a random *d*-dimensional output vector. For the variational classifier q(y|z) any classifier network can be used, including just a linear softmax classifier as done in these experiments. For the variational conditional marginal q(z|y) it helps to use the same distribution as output by the classifier. For the simple unit variance Gaussian encoding we used in these experiments, this requires learning just *d* parameters per class. For ease of implementation, this can be represented as a single dense linear layer mapping from a one-hot representation of the labels to the *d*-dimensional output, interpreted as the mean of the corresponding class marginal.

In this setup the CEB loss takes a particularly simple form:

(8) $$\begin{array}{cc} \; & E\left[w_{y}\cdot \left(f\left(x\right)+\epsilon \right)-\log\sum_{y^{\prime }}e^{w_{y^{\prime }}\cdot \left(f\left(x\right)+\epsilon \right)}-\frac{e^{-\rho }}{2}\left(f\left(x\right)-\mu_{y}\right)\left(f\left(x\right)-\mu_{y}+2\epsilon \right)\right]. \end{array}$$

The first two terms of Equation (8) are the usual softmax classifier loss, but acting on our stochastic representation z=f(x)+ϵ, which is simply the output of our encoder network f(x) with additive Gaussian noise. The $w_{y}$ is the *y*th row of weights in the final linear layer outputting the logits. $\mu_{y}$ are the learned class conditional means for our marginal. ϵ are standard normal draws from an isotropic unit variance Gaussian with the same dimension as our encoding f(x). The last term of Equation (8) is a stochastic sampling of the KL divergence between our encoder likelihood and the class conditional marginal likelihood. ρ controls the strength of the bottleneck and can vary on the whole real line. As ρ→∞ the bottleneck is turned off. In practice we find that ρ values near but above 0 tend to work best for modest size models, with the tendency for the best ρ to approach 0 as the model capacity increases. Notice that in expectation the second term in the loss is ${\left(f\left(x\right)-\mu_{y}\right)}^{2}$, which encourages the learned means $\mu_{y}$ to converge to the average of the representations of each element in the class. During testing we use the mean encodings and remove the stochasticity.

In its simplest form, training a CEB classifier amounts to injecting Gaussian random noise in the penultimate layer and learning estimates of the class-averaged output of that layer. In Appendix B we show simple modifications to the TPU-compatible ResNet implementation available on GitHub from the Google TensorFlow Team [18] that produce the same core ResNet50 models we use for our ImageNet experiments.

### 2.3. Consistent Classifier

An alternative classifier to the standard linear layer described in Section 2.2 performs the Bayesian inversion on the true class-conditional marginal:

(9) $$\begin{array}{c} p\left(y|z\right)=\frac{p(z|y)p(y)}{\sum_{y^{\prime }}p\left(z|y^{\prime }\right)p\left(y^{\prime }\right)}. \end{array}$$

Substituting q(z|y) and using the empirical distribution over labels, we can define our variational classifier as:

(10) $$\begin{array}{c} q\left(y|z\right)\equiv {\mathrm{softmax}}_{y}\left(q\left(z|y\right)p\left(y\right)\right) \end{array}$$

In the case that the labels are uniformly distributed, that further simplifies to $q\left(y|z\right)\equiv {\mathrm{softmax}}_{y}\left(q\left(z|y\right)\right)$. We call this the *consistent classifier* because it is Bayes-consistent with the variational conditional marginal. This is in contrast to the standard feed-forward classifier, which may choose to classify a region of the latent space differently from the highest density class given by the conditional marginal.

### 2.4. Adversarial Attacks and Defenses

#### 2.4.1. Attacks

The first adversarial attacks were proposed in Szegedy et al. [1], Goodfellow et al. [19]. Since those seminal works, an enormous variety of attacks has been proposed (Carlini and Wagner [2], Kurakin et al. [4], Madry et al. [5], Kurakin et al. [20], Moosavi-Dezfooli et al. [21], Eykholt et al. [22], Baluja and Fischer [23], etc.). In this work, we will primarily consider the Projected Gradient Descent (PGD) attack [5], which is a multi-step variant of the early Fast Gradient Method [19]. The attack can be viewed as having four parameters—*p*, the norm of the attack (typically 2 or *∞*), ϵ, the radius the the *p*-norm ball within which the attack is permitted to make changes to an input, *n*, the number of gradient steps the adversary is permitted to take, and $\epsilon_{i}$, the per-step limit to modifications of the current input. In this work, we consider L${}_{2}$ and L${}_{\infty }$ attacks of varying ϵ and *n*, and with $\epsilon_{i}=\frac{4}{3}\frac{\epsilon }{n}$.

#### 2.4.2. Defenses

A common defense for adversarial examples is adversarial training. Adversarial training was originally proposed in Szegedy et al. [1], but was not practical until the Fast Gradient Method was introduced. It has been studied in detail, with varied techniques [5,20,24,25]. Adversarial training can clearly be viewed as a form of data augmentation [26], where instead of using some fixed set of functions to modify the training examples, we use the model itself in combination with one or more adversarial attacks to modify the training examples. As the model changes, the distribution of modifications changes as well. However, unlike with non-adversarial data augmentation techniques, such as AutoAugment (AutoAug) [9], adversarial training techniques considered in the literature so far cause substantial reductions in accuracy on clean test sets. For example, the CIFAR10 model described in Madry et al. [5] gets 95.5% accuracy when trained normally, but only 87.3% when trained on L${}_{\infty }$ adversarial examples. More recently, Xie et al. [25] adversarially trains ImageNet models with impressive robustness to targeted PGD L${}_{\infty }$ attacks, but at only 62.32% accuracy on the non-adversarial test set, compared to 78.81% accuracy for the same model trained only on clean images.

### 2.5. Common Corruptions

The Common Corruptions Benchmark [8] offers a test of model robustness to common image processing pipeline corruptions. ImageNet-C modifies the ImageNet test set with the 15 corruptions applied at five different strengths. Within each corruption type we evaluate the average error at each of the five levels ($E_{c}=\frac{1}{5}\sum_{s=1}^{5}E_{cs}$). To summarize the performance across all corruptions, we report both the average corruption error ($\mathrm{avg}=\frac{1}{15}\sum_{c}E_{c}$) and the *Mean Corruption Error* (mCE) [8]:

(11) $$\mathrm{mCE}=\frac{1}{15}\sum_{c}\frac{\sum_{s=1}^{5}E_{cs}}{\sum_{s=1}^{5}E_{cs}^{\mathrm{AlexNet}}}.$$

The mCE weights the errors on each task against the performance of a baseline AlexNet model. Slightly different pipelines have been used for the ImageNet-C task [10]. In this work we used the AlexNet normalization numbers and data formulation from Yin et al. [11].

### 2.6. Natural Adversarial Examples

The ImageNet-A Benchmark [27] is a dataset of 7500 naturally-occurring “adversarial” examples across 200 ImageNet classes. The images exploit commonly-occurring weaknesses in ImageNet models, such as relying on textures often seen with certain class labels.

### 2.7. Calibration

One approach to estimating a model’s robustness is to look at how well *calibrated* the model is. The *Expected Calibration Error* (ECE) [28] gives an intuitive metric of calibration:

(12) $$\begin{array}{c} ECE\equiv \sum_{s=1}^{S}\frac{|B_{s}|}{N}\left|\mathrm{acc}\left(B_{s}\right)-\mathrm{conf}\left(B_{s}\right)\right|, \end{array}$$

where *S* is the number of confidence bins (30 in our experiments), *N* is the number of examples (50,000 for ImageNet and for each ImageNet-C corruption), $|B_{s}|$ is the number of examples in the *s*th bin, $\mathrm{acc}(B_{s})$ is the mean accuracy in the *s*th bin, and $\mathrm{conf}(B_{s})$ is the mean confidence of the model’s predictions in the *s*th bin. The ECE ranges between 0 and 1. A perfectly calibrated model would have an ECE of 0. See Ovadia et al. [29] for further details.

## 3. Results

### 3.1. CIFAR10 Experiments

**Figure 1:**
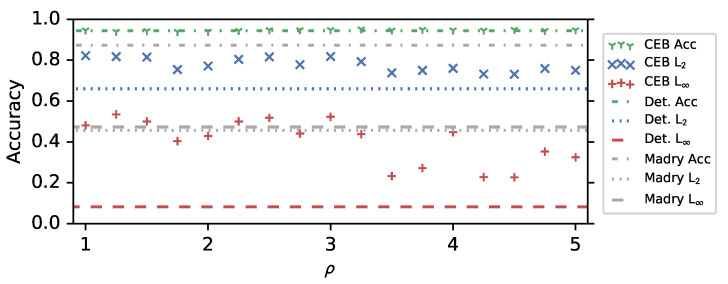
Conditional Entropy Bottleneck (CEB) ρ vs. test set accuracy, and L${}_{2}$ and L${}_{\infty }$ Projected Gradient Descent (PGD) adversarial attacks on CIFAR10. *None of the CEB models is adversarially trained.*

We trained a set of 25 28×10 Wide ResNet (WRN) CEB models on CIFAR10 at ρ∈[−1,−0.75,…,5], as well as a deterministic baseline. They trained for 1500 epochs, lowering the learning rate by a factor of 0.3 after 500, 1000, and 1250 epochs. This long training regime was due to our use of the original AutoAug policies, which requires longer training. The only additional modification we made to the basic 28×10 WRN architecture was the removal of all Batch Normalization [30] layers. Every small CIFAR10 model we have trained with Batch Normalization enabled has had substantially worse robustness to L${}_{\infty }$ PGD adversaries, even though typically the accuracy is much higher. For example, 28×10 WRN CEB models rarely exceeded more than 10% adversarial accuracy. However, it was always still the case that lower values of ρ gave higher robustness. As a baseline comparison, a deterministic 28×10 WRN with BatchNorm, trained with AutoAug reaches 97.3% accuracy on clean images, but 0% accuracy on L${}_{\infty }$ PGD attacks at ϵ=8 and n=20. Interestingly, that model was noticeably more robust to L${}_{2}$ PGD attacks than the deterministic baseline without BatchNorm, getting 73% accuracy compared to 66%. However, it was still much weaker than the CEB models, which get over 80% accuracy on the same attack (Figure 1). Additional training details are in Appendix A.1.

Figure 1 demonstrates the adversarial robustness of CEB models to both targeted L${}_{2}$ and L${}_{\infty }$ attacks. The CEB models show a marked improvement in robustness to L${}_{2}$ attacks compared to an adversarially-trained baseline from Madry et al. [5] (denoted Madry). The attack parameters were selected to be about equally difficult for the adversarially-trained WRN 28×10 model from Madry et al. [5] (grey dashed and dotted lines in Figure 1). The deterministic baseline (Det.) only gets 8% accuracy on the L${}_{\infty }$ attacks, but gets 66% on the L${}_{2}$ attack, substantially better than the 45.7% of the adversarially-trained model, which makes it clear that the adversarially-trained model failed to generalize in any reasonable way to the L${}_{2}$ attack. The CEB models are always substantially more robust than Det., and many of them outperform Madry even on the L${}_{\infty }$ attack the Madry model was trained on, but for both attacks there is a clear general trend toward more robustness as ρ decreases. Finally, the CEB and Det. models all reach about the same accuracy, ranging from 93.9% to 95.1%, with Det. at 94.4%. In comparison, Madry only gets 87.3%.

**Figure 2:**
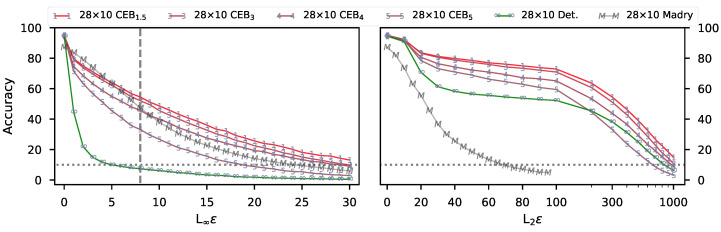
Untargeted adversarial attacks on CIFAR10 models showing both strong robustness to PGD L${}_{2}$ and L${}_{\infty }$ attacks, as well as good test accuracy of up to 95.1%. (**Left**): Accuracy on untargeted L${}_{\infty }$ attacks at different values of ε for all 10,000 test set examples. (**Right**): Accuracy on untargeted L${}_{2}$ attacks at different values of ε. Note the switch to log scale on the x axis at L${}_{2}\epsilon =100$. 28×10 indicates the Wide ResNet size. CEB${}_{x}$ indicates a CEB model trained at ρ=x. Madry is the adversarially-trained model from Madry et al. [5] (values provided by Aleksander Madry). *None of the CEB models is adversarially-trained.*

Figure 2 shows the robustness of five of those models to PGD attacks as ϵ is varied. We selected the four CEB models to represent the most robust models across most of the range of ρ we trained. All values in the figure are collected at 20 steps of PGD. The Madry model [5] was trained with 7 steps of L${}_{\infty }$ PGD at ε=8 (grey dashed line in the figure). All of the CEB models with ρ≤4 outperform Madry across most of the values of ϵ, even though they were not adversarially-trained. It is interesting to note that the Det. model eventually outperforms the CEB${}_{5}$ model on L${}_{2}$ attacks at relatively high accuracies. This result indicates that the CEB${}_{5}$ model may be under-compressed.

Of the 25 CEB models we trained, only the models with ρ≥1 successfully trained. The remainder collapsed to chance performance. This is something we observe on all datasets when training models that are too low capacity. Only by increasing model capacity does it become possible to train at low ρ. Note that this result is predicted by the theory of the onset of learning in IB and its relationship to model capacity from Wu et al. [31].

We additionally tested two models (ρ=0 and ρ=5) on the CIFAR10 Common Corruptions test sets. At the time of training, we were unaware that AutoAug’s default policies for CIFAR10 contain brightness and contrast augmentations that amount to training on those two corruptions from Common Corruptions (as mentioned in Yin et al. [11]), so our results are not appropriate for direct comparison with other results in the literature. However, they still allow us to compare the effect of bottlenecking the information between the two models. The ρ=5 model reached an mCE of 61.2. The ρ=0 model reached an mCE of 52.0, which is a dramatic relative improvement. Note that the mCE is computed relative to a baseline model. We use the baseline model from Yin et al. [11].

### 3.2. ImageNet Experiments

To demonstrate CEB’s ability to improve robustness, we trained four different ResNet architectures on ImageNet at 224×224 resolution, with and without AutoAug, using three different objective functions, and then tested them on ImageNet-C, ImageNet-A, and targeted PGD attacks.

**Figure 3:**
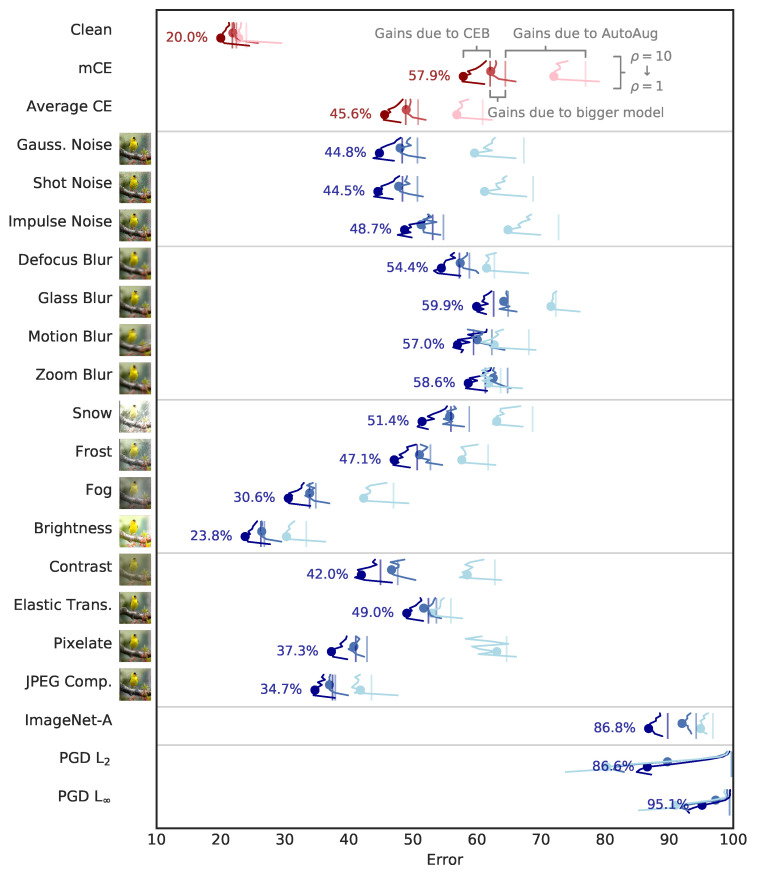
Summary of the ResNet50 ImageNet-C experiments. Lower is better in all cases. In the main part of the figure (in blue), the average errors across corruption magnitude are shown for 33 different networks for each of the labeled Common Corruptions, ImageNet-A, and targeted PGD attacks. The networks come in paired sets, with the vertical lines denoting the baseline XEnt network’s performance, and then in the corresponding color the errors for each of 10 different CEB networks are shown with varying ρ=[1,2,…,10], arranged from 10 at the top to 1 at the bottom. The light blue lines indicate ResNet50 models trained without AutoAug. The blue lines show the same network trained with AutoAug. The dark blue lines show ResNet50 AutoAug networks that were made twice as wide. For these models, we display cCEB rather than CEB, which gave qualitatively similar but slightly weaker performance. The figure separately shows the effects of data augmentation, enlarging the model, and the additive effect of CEB on each model. At the top in red are shown the same data for three summary statistics. clean denotes the clean top-1 errors of each of the networks. mCE denotes the AlexNet regularized average corruption errors. avg shows an equally-weighted average error across all common corruptions. The dots denote the value for each CEB network and each corruption at $\rho^{*}$, the optimum ρ for the network as measured in terms of clean error. The values at these dots and the baseline values are given in detail in Table 1. Figure 4 show the same data for the ResNet152 models.

**Figure 4:**
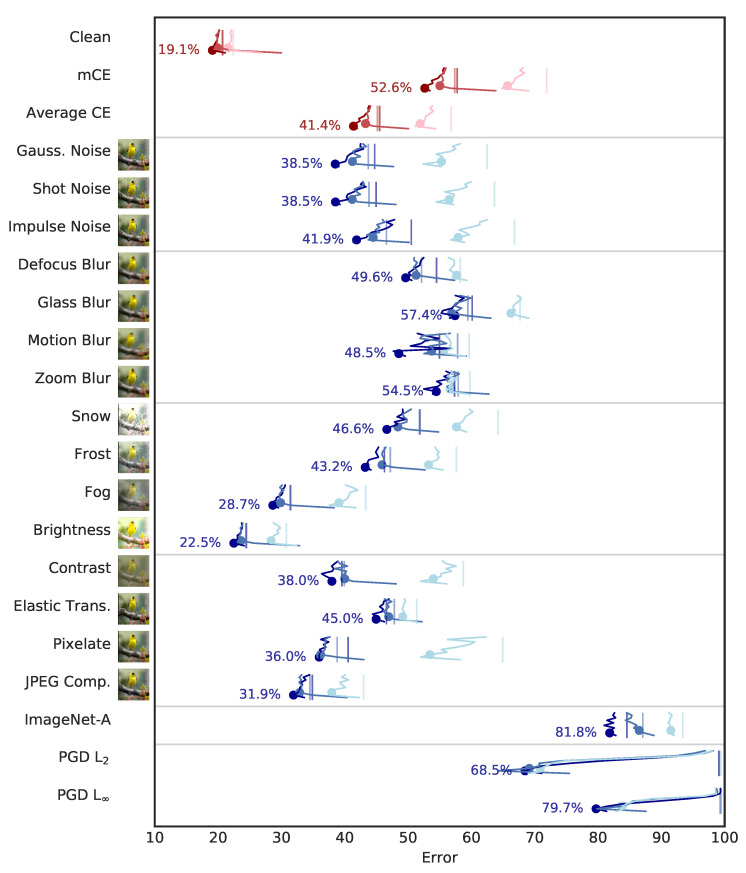
Replication of Figure 3 but for ResNet152. Lower is better in all cases. The light blue lines indicate ResNet152 models trained without AutoAug. The blue lines show the same network trained with AutoAug. The dark blue lines show ResNet152 AutoAug networks that were made twice as wide. As in Figure 3, we show the cCEB models for the largest network to reduce visual clutter. The deeper model shows marked improvement across the board compared to ResNet50, but the improvements due to CEB and cCEB are even more striking. Notice in particular the adversarial robustness to L${}_{\infty }$ and L${}_{2}$ PGD attacks for the CEB models over the XEnt baselines. The L${}_{\infty }$ baselines all have error rates above 99%, so they are to the right edge of the figure. See Table 1 for details of the best-performing models, which correspond to the dots in this figure.

As a simple baseline we trained ResNet50 with no data augmentation using the standard cross-entropy loss (XEnt). We then trained the same network with CEB at ten different values of ρ=(1,2,…,10). AutoAug [9] has previously been demonstrated to improve robustness markedly on ImageNet-C, so next we trained ResNet50 with AutoAug using XEnt. We similarly trained these AutoAug ResNet50 networks using CEB at the same ten values of ρ. ImageNet-C numbers are also sensitive to the model capacity. To assess whether CEB can benefit larger models, we repeated the experiments with a modified ResNet50 network where every layer was made twice as wide, training an XEnt model and ten CEB models, all with AutoAug. To see if there is any additional benefit or cost to using the consistent classifier (Section 2.3), we took the same wide architecture using AutoAug and trained ten consistent classifier CEB (cCEB) models. Finally, we repeated all of the previous experiments using ResNet152: XEnt and CEB models without AutoAug; with AutoAug; with AutoAug and twice as wide; and cCEB with AutoAug and twice as wide. All other hyperparameters (learning rate schedule, L${}_{2}$ weight decay scale, etc.) remained the same across all models. All of those hyperparameters where taken from the ResNet hyperparameters given in the AutoAug paper. In total we trained 86 ImageNet models: 6 deterministic XEnt models varying augmentation, width, and depth; 60 CEB models additionally varying ρ; and 20 cCEB models also varying ρ. The results for the ResNet50 models are summarized in Figure 3. For ResNet152, see Figure 4. See Table 1 for detailed results across the matrix of experiments. Additional experimental details are given in Appendix A.2.

The CEB models highlighted in Figure 3 and Figure 4 and Table 1 were selected by cross validation. These were values of ρ that gave the best *clean* test set accuracy. Despite being selected for classical generalization, these models also demonstrate a high degree of robustness on both average- and worst-case perturbations. In the case that more than one model gets the same test set accuracy, we choose the model with the lower ρ, since we know that lower ρ correlates with higher robustness. The only model where we had to make this decision was for ResNet152 with AutoAug, where five models all were within 0.1% of each other, so we chose the ρ=3 model, rather than ρ∈{5…8}.

#### 3.2.1. Accuracy, ImageNet-C, and ImageNet-A

Increasing model capacity and using AutoAug have positive effects on classification accuracy, as well as on robustness to ImageNet-C and ImageNet-A, but for all three classes of models CEB gives substantial additional improvements. cCEB gives a small but noticeable additional gain for all three cases (except indistinguishable performance compared to CEB on ImageNet-A with the wide ResNet152 architecture), indicating that enforcing variational consistency is a reasonable modification to the CEB objective. In Table 1 we can see that CEB’s relative accuracy gains increase as the architecture gets larger, from gains of 1.2% for ResNet50 and ResNet152 without AutoAug, to 1.6% and 1.8% for the consistent wide models with AutoAug. This indicates that even larger relative gains may be possible when using CEB to train larger architectures than those considered here. We can also see that for the XEnt 152x2 and 152 models, the smaller model (152) actually has better mCE and equally good top-1 accuracy, indicating that the wider model may be overfitting, but the 152x2 CEB and cCEB models substantially outperform both of them across the board. cCEB gives a noticeable boost over CEB for clean accuracy and mCE in both wide architectures.

#### 3.2.2. Targeted PGD Attacks

We tested on the random-target version of the PGD L${}_{2}$ and L${}_{\infty }$ attacks [4]. The L${}_{\infty }$ attack used ϵ=16, n=10, and $\epsilon_{i}=2$, which is considered to be a strong attack still [25]. The L${}_{2}$ attack used ϵ=200, n=10, and $\epsilon_{i}=220$. Those parameters were chosen by attempting to match the baseline XEnt ResNet50 without AutoAug model’s performance on the L${}_{\infty }$ attack—the performance of the CEB models were not considered when selecting the L${}_{2}$ attack strength. Interestingly, for the PGD attacks, AutoAug was detrimental—the ResNet50 models without AutoAug were substantially more robust than those with AutoAug, and the ResNet152 models without AutoAug were nearly as robust as the AutoAug and wide models, in spite of having much worse test set accuracy. The ResNet50 CEB models show a dramatic improvement over the XEnt model, with top-1 accuracy increasing from 0.3% to 19.8% between the XEnt baseline without AutoAug and the corresponding ρ=4 CEB model, a relative increase of 66 times. Interestingly, the CEB ResNet50 models *without* AutoAug are much more robust to the adversarial attacks than the AutoAug and wide ResNet50 models. As with the accuracy results above, the robustness gains due to CEB increase as model capacity increases, indicating that further gains are possible.

#### 3.2.3. Calibration and ImageNet-C

**Figure 5:**
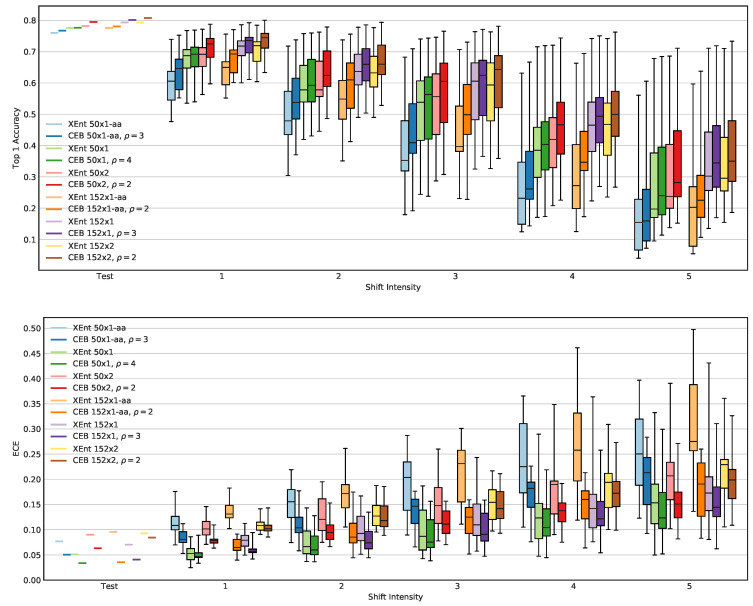
Comparison of accuracy and Expected Calibration Error (ECE) between Xent baseline models and corresponding CEB models at the value of ρ that gives the closest accuracy to the XEnt baseline. Higher is better for accuracy; lower is better for ECE. The box plots show the minimum, 25th percentile, mean, 75th percentile, and maximum values across the 15 different ImageNet-C corruptions for the given shift intensity. XEnt baseline models are always the lighter color, with the corresponding CEB model having the darker color.

Following the experimental setup in Reference [29], in Figure 5 we compare accuracy and ECE on ResNet models for both the clean ImageNet test set and the collection of 15 ImageNet-C corruptions at each of the five different corruption intensities. It is easy to see in the figure that the CEB models always have superior mean accuracy and ECE for all six different sets of test sets.

Because accuracy can have a strong impact on ECE, we use a different model selection procedure than in the previous experiments. Rather than selecting the CEB model with the highest accuracy, we instead select the CEB model with with the *closest* accuracy to the corresponding XEnt model. This resulted in selecting models with lower ρ than in the previous experiments for four out of the six CEB model classes. We note that by selecting models with lower ρ (which are more compressed), we see more dramatic differences in ECE, but even if we select the CEB models with highest accuracy as in the previous experiments, all six CEB models outperform the corresponding XEnt baselines on all six different sets of test sets.

## 4. Conclusions

The Conditional Entropy Bottleneck (CEB) provides a simple mechanism to improve robustness of image classifiers. We have shown a strong trend toward increased robustness as ρ decreases in the standard 28×10 Wide ResNet model on CIFAR10, and that this increased robustness does not come at the expense of accuracy relative to the deterministic baseline. We have shown that CEB models at a range of ρ outperform an adversarially-trained baseline model, even on the attack the adversarial model was trained on, and have incidentally shown that the adversarially-trained model generalizes to at least one other attack *less well* than a deterministic baseline. Finally, we have shown that on ImageNet, CEB provides substantial gains over deterministic baselines in validation set accuracy, robustness to Common Corruptions, Natural Adversarial Examples, and targeted Projected Gradient Descent attacks, and gives large improvements to model calibration, all without any change to the inference architecture. We hope these empirical demonstrations inspire further theoretical and practical study of the use of bottlenecking techniques to encourage improvements to both classical generalization and robustness.

## Figures and Tables

**Table 1:** Baseline and cross-validated CEB values for the ImageNet experiments. **cCEB** uses the consistent classifier. **XEnt** is the baseline cross entropy objective. “**-aa**” indicates AutoAug is not used during training. “**x2**” indicates the ResNet architecture is twice as wide. The CEB values reported here are denoted with the dots in Figure 3 and Figure 4. Lower values are better in all cases, and the lowest value for each architecture is shown in bold. All values are percentages.

Architecture	ResNet152x2			ResNet152		ResNet152-aa		ResNet50x2			ResNet50		ResNet50-aa	
**Objective**	**cCEB**	**CEB**	**XEnt**	**CEB**	**XEnt**	**CEB**	**XEnt**	**cCEB**	**CEB**	**XEnt**	**CEB**	**XEnt**	**CEB**	**XEnt**
ρ	2	2	NA	3	NA	3	NA	4	3	NA	6	NA	4	NA
Clean	**19.1**	19.3	20.7	**19.9**	20.7	**21.6**	22.4	**20.0**	20.2	21.8	**21.9**	22.5	**22.8**	24.0
mCE	**52.6**	53.2	57.8	**55.0**	57.4	**65.7**	71.9	57.9	**57.8**	62.0	**62.1**	64.4	**72.0**	77.0
Average CE	**41.4**	41.8	45.5	**43.3**	45.2	**51.9**	56.8	45.6	**45.5**	48.9	**49.0**	50.8	**56.9**	60.9
Gauss. Noise	**38.5**	40.1	44.7	**41.2**	43.7	**55.3**	62.5	44.8	**43.9**	48.3	**48.0**	50.7	**59.6**	67.3
Shot Noise	**38.5**	40.3	45.0	**41.2**	43.8	**56.5**	63.7	44.5	**43.9**	48.4	**47.8**	50.7	**61.2**	68.8
Impulse Noise	**41.9**	43.6	50.5	**44.5**	46.6	**57.9**	66.8	48.7	**48.1**	53.1	**51.3**	54.8	**64.8**	72.7
Defocus Blur	49.6	**48.8**	54.5	**51.3**	52.1	**57.7**	58.3	54.4	**54.2**	57.3	**57.4**	58.8	**61.5**	62.7
Glass Blur	57.4	**56.7**	60.1	**56.9**	59.4	**66.2**	67.7	**59.9**	61.0	62.6	**64.2**	64.9	**71.5**	72.3
Motion Blur	**48.5**	51.4	55.0	**53.7**	57.8	**55.6**	59.7	57.0	**56.6**	59.5	**60.0**	62.3	**62.7**	68.1
Zoom Blur	**54.5**	54.7	57.3	**56.8**	57.9	**56.6**	59.8	58.6	**58.0**	61.3	**62.5**	64.8	**61.8**	63.7
Snow	**46.6**	**46.6**	51.9	**48.4**	51.8	**57.6**	64.2	51.4	**50.9**	55.9	**55.7**	58.8	**63.1**	68.7
Frost	**43.2**	43.9	46.3	**45.9**	47.2	**53.2**	57.6	**47.1**	**47.1**	50.7	**51.0**	52.7	**57.6**	61.7
Fog	**28.7**	**28.7**	31.4	**29.9**	31.5	**39.1**	43.3	30.6	**30.2**	33.9	**33.9**	34.8	**42.3**	47.0
Brightness	**22.5**	22.6	24.5	**23.6**	24.4	**28.4**	30.8	**23.8**	24.1	26.3	**26.4**	26.8	**30.3**	33.4
Contrast	38.0	**37.7**	39.5	40.0	**39.9**	**54.0**	58.7	**42.0**	42.4	44.9	**46.7**	47.6	**58.5**	62.8
Elastic Trans.	**45.0**	45.3	46.6	**46.9**	47.8	**49.2**	51.4	49.0	**48.8**	52.4	**51.7**	53.7	**53.0**	56.0
Pixelate	36.0	**35.2**	40.5	**36.2**	38.8	**53.4**	64.9	**37.3**	37.9	41.1	**40.8**	42.8	**63.1**	64.6
JPEG Comp.	31.9	**31.8**	34.9	**32.9**	34.5	**38.0**	43.0	**34.7**	35.1	37.4	**37.0**	37.9	**41.8**	43.5
ImageNet-A	**81.8**	82.0	84.6	**86.5**	87.1	**91.5**	93.4	**86.8**	88.1	89.8	**92.0**	94.2	**94.9**	96.8
PGD L${}_{2}$	68.5	**68.0**	99.1	**69.1**	99.2	**70.9**	99.4	86.6	**84.5**	99.8	**89.7**	99.7	**80.2**	99.7
PGD L${}_{\infty }$	79.7	**79.3**	99.3	**83.8**	99.4	**83.8**	99.4	95.1	**93.2**	99.4	**97.3**	99.4	**91.0**	99.5

## Appendix A. Experiment Details

Here we give additional technical details for the CIFAR10 and ImageNet experiments.

### Appendix A.1. CIFAR10 Experiment Details

We trained all of the models using Adam [32] at a base learning rate of $10^{-3}$. We lowered the learning rate three times by a factor of 0.3 each time. The only additional trick to train the CIFAR10 models was to start with ρ=100, anneal down to ρ=10 over 2 epochs, and then anneal to the target ρ over one epoch once training exceeded a threshold of 20%. This jump-start method is inspired by experiments on VIB in Wu et al. [31]. It makes it much easier to train models at low ρ, and appears to not negatively impact final performance.

### Appendix A.2. ImageNet Experiment Details

We follow the learning rate schedule for the ResNet 50 from Cubuk et al. [9], which has a top learning rate of 1.6, trains for 270 epochs, and drops the learning rate by a factor of 10 at 90, 180, and 240 epochs. The only difference for all of our models is that we train at a batch size of 8192 rather than 4096. Similar to the CIFAR10 models, in order to ensure that the ImageNet models train at low ρ, we employ a simple jump-start. We start at ρ=100 and anneal down to the target ρ over 12,000 steps. The first learning rate drop occurs a bit after 14,000 steps. Also similar to the CIFAR10 28×10 WRN experiments, none of the models we trained at ρ=0 succeeded, indicating that ResNet50 and wide ResNet50 both have insufficient capacity to fully learn ImageNet. We were able to train ResNet152 at ρ=0, but only by disabling L${}_{2}$ weight decay and using a slightly lower learning rate. Since that involved additional hyperparameter tuning, we do not report those results here, beyond noting that it is possible, and that those models reached top-1 accuracy around 72%.

## Appendix B. CEB Example Code

In Listing 1 Listing 2 and Listing 3 we give annotated code changes needed to make ResNet CEB models, based on the TPU-compatible ResNet implementation from the Google TensorFlow Team [18].

**Listing 1:** Modifications to the model.py file.

*#In model.py*:
defresnet_v1_generator(block_fn, layers, num_classes,…):
def model(inputs, is_training):
* # Build the ResNet model as normal up to the following lines:*
inputs = tf.reshape(
inputs, [−1, 2048 if block_fn is bottleneck_block else 512])
* # Now, instead of the final dense layer, just~return inputs,*
*# which for ResNet50 models is a [batch_size, 2048] tensor.*
return inputs

**Listing 2:** Modification to the head of resnet_main.py.

*# In resnet_main.py add the following imports and functions:*
import tensorflow_probability as tfp
tfd = tfp.distributions

defezx_dist(x):
*"""Builds the encoder distribution, e(z|x)."""*
dist = tfd.MultivariateNormalDiag(loc = x)
return dist

defbzy_dist(y, num_classes = 1000, z_dims = 2048):
*"""Builds the backwards distribution, b(z|y)."""*
y_onehot = tf.one_hot(y, num_classes)
mus =| tf.layers.dense(y_onehot, z_dims, activation = None)
dist = tfd.MultivariateNormalDiag(loc = mus)
return dist

defcyz_dist(z, num_classes = 1000):
*"""Builds the classifier distribution, c(y|z)."""*
*# For the classifier, we~are using exactly the same dense layer*
*# initialization as was used for the final layer that we removed*
*# from model.py.*
logits = tf.layers.dense(
z, num_classes, activation = None,
kernel_initializer=tf.random_normal_initializer(stddev = 0.01))
return tfd.Categorical(logits=logits)

deflerp(global_step, start_step, end_step, start_val, end_val):
* """Utility function to linearly interpolate two values."""*
interp = (tf.cast(global_step - start_step, tf.float32)
/ tf.cast(end_step - start_step, tf.float32))
interp = tf.maximum(0.0, tf.minimum(1.0, interp))
return start_val ∗ (1.0 - interp) + end_val ∗ interp

**Listing 3:** Modifications to resnet_model_fn in resnet_main.py.

*# Still in resnet_main.py, modify resnet_model_fn as follows:*
defresnet_model_fn(features, labels, mode, params):
*# Nothing changes until after the definition of build_network:*
def build_network():
*# Elided, unchanged implementation of build_network.*

if params['precision'] == 'bfloat16':
*#build_network now returns the pre-logits, so~we'll change*
*# the variable name from logits to net.*
with tf.contrib.tpu.bfloat16_scope():
net = build_network()
net = tf.cast(net, tf.float32)
elif params['precision'] == 'float32':
net = build_network()

*# Get the encoder, e(z|x):*
with tf.variable_scope('ezx', reuse=tf.AUTO_REUSE):
ezx = ezx_dist(net)
*# Get the backwards encoder, b(z|y):*
with tf.variable_scope('bzy', reuse=tf.AUTO_REUSE):
bzy = bzy_dist(labels)

*# Only sample z during training. Otherwise, just~pass through*
*# the mean value of the encoder.*
if mode == tf.estimator.ModeKeys.TRAIN:
z = ezx.sample()
else:
z = ezx.mean()

*#Get the classifier, c(y|z):*
with tf.variable_scope('cyz', reuse=tf.AUTO_REUSE):
cyz = cyz_dist(z, params)

*# cyz.logits is the same as what the unmodified ResNet model would return.*
logits = cyz.logits

*# Compute the individual conditional entropies:*
hzx = −ezx.log_prob(z) # H(Z|X)
hzy = −bzy.log_prob(z) # H(Z|Y) (upper bound)
hyz = −cyz.log_prob(labels) # H(Y|Z) (upper bound)
*# I(X;Z|Y) = −H(Z|X) + H(Z|Y)*
* # >= −hzx + hzy =: Rex, the~residual information.*
rex = −hzx + hzy

rho = 3.0 # You should make this a hyperparameter.
rho_to_gamma = lambda rho: 1.0 / np.exp(rho)
gamma = tf.cast(rho_to_gamma(rho), tf.float32)

*# Get the global step now, so~that we can adjust rho dynamically.*
global_step = tf.train.get_global_step()

anneal_rho = 12,000 *# You should make this a hyperparameter.*
if anneal_rho > 0:
*# Anneal rho from 100 down to the target rho*
*# over the first anneal_rho steps.*
gamma = lerp(global_step, 0, aneal_rho,
rho_to_gamma(100.0), gamma)

*# Replace all the softmax cross-entropy loss computation with the following line:*
loss = tf.reduce_mean(gamma ∗ rex + hyz)
*# The rest of resnet_model_fn can remain unchanged.*
